# Comparative assessment of the effects of DREADDs and endogenously expressed GPCRs in hippocampal astrocytes on synaptic activity and memory

**DOI:** 10.3389/fncel.2023.1159756

**Published:** 2023-03-27

**Authors:** Sophie H. Lee, Aline Mak, Mark H. G. Verheijen

**Affiliations:** ^1^Department of Molecular and Cellular Neurobiology, Center for Neurogenomics and Cognitive Research, Amsterdam Neuroscience, Vrije Universiteit Amsterdam, Amsterdam, Netherlands; ^2^Research Master’s Programme Brain and Cognitive Sciences, University of Amsterdam, Amsterdam, Netherlands

**Keywords:** glia, tripartite synapse, signal transduction, signaling pathways, behavior

## Abstract

Designer Receptors Exclusively Activated by Designer Drugs (DREADDs) have proven themselves as one of the key *in vivo* techniques of modern neuroscience, allowing for unprecedented access to cellular manipulations in living animals. With respect to astrocyte research, DREADDs have become a popular method to examine the functional aspects of astrocyte activity, particularly G-protein coupled receptor (GPCR)-mediated intracellular calcium (Ca^2+^) and cyclic adenosine monophosphate (cAMP) dynamics. With this method it has become possible to directly link the physiological aspects of astrocytic function to cognitive processes such as memory. As a result, a multitude of studies have explored the impact of DREADD activation in astrocytes on synaptic activity and memory. However, the emergence of varying results prompts us to reconsider the degree to which DREADDs expressed in astrocytes accurately mimic endogenous GPCR activity. Here we compare the major downstream signaling mechanisms, synaptic, and behavioral effects of stimulating Gq-, Gs-, and Gi-DREADDs in hippocampal astrocytes of adult mice to those of endogenously expressed GPCRs.

## 1. Introduction

While astrocytes were traditionally seen as simple, supportive cells within the brain, the advent of Ca^2+^ imaging technologies in the 1990s led to the understanding that astrocytes exhibit a non-electrical form of excitability mediated by intracellular Ca^2+^ signaling (Cornell-Bell et al., 1990; Dani et al., 1992). Following this discovery, it was shown that astrocytes are intricately associated with neurons at the synapse, forming what is often termed the “tripartite synapse”: a functional unit consisting of pre- and post-synaptic neurons and peri-synaptic astrocyte processes (PAPs) (Araque et al., 1999; Papouin et al., 2017). Although the extent of gliotransmission remains debated (Bazargani and Attwell, 2016; Fiacco and McCarthy, 2018; Savtchouk and Volterra, 2018; Durkee et al., 2019), astrocytes are known to express a plethora of receptors which are also present on neurons, and a multitude of research now illustrates the contribution of astrocytes to synaptic activity and behavior, through dynamic interactions with neurons (Durkee and Araque, 2019; Lawal et al., 2022; Lyon and Allen, 2022).

G-protein coupled receptors (GPCRs) are a major family of metabotropic receptors abundantly expressed on both neurons and astrocytes. The presence of GPCRs on astrocytes is thought to allow these cells to receive and respond to local synaptic activity and environmental cues such as hormones and extracellular matrix molecules (Roux and Cottrell, 2014; Durkee and Araque, 2019; Kofuji and Araque, 2021). Astrocytic GPCRs can be activated by neurotransmitters such as glutamate, gamma-aminobutyric acid (GABA), adenosine triphosphate (ATP) and endocannabinoids, triggering a variety of downstream signaling pathways by elevation of intracellular Ca^2+^ (Durkee and Araque, 2019) or cyclic adenosine monophosphate (cAMP) (Zhou et al., 2019). There is a growing body of evidence suggesting that astrocytic GPCRs are instrumental in mediating memory processes (Santello et al., 2019; Kol and Goshen, 2021; Lyon and Allen, 2022), although much of this research utilizes artificial rather than endogenously expressed GPCRs. Indeed, methodological challenges in studying endogenous GPCR activity in astrocytes *in vivo* has limited our understanding of how they impact cognition. For instance, while a wide variety of techniques are available to study the function of endogenous GPCRs in astrocytes, assessing behavioral outcomes of astrocytic GPCR modulation often requires sacrificing the physiological plausibility of the intervention (Xie et al., 2015).

Chemogenetics has become a popular remedy to the difficulties in assessing the behavioral consequences of astrocytic GPCR activity. This methodology allows for the activity of genetically engineered proteins to be altered by a biologically inert chemical ligand (Roth, 2016). Designer Receptors Exclusively Activated by Designer Drugs (DREADDs) (Armbruster et al., 2007) are the most commonly used chemogenetic tool for this purpose. DREADDs aim to mimic endogenous GPCR activity (Atasoy and Sternson, 2018), and confer a number of advantages to other techniques, including increased physiological plausibility and access to behavioral effects (Xie et al., 2015). However, studies using this method to uncover the role of astrocytic GPCRs in synaptic activity and behavior have produced a number of varying results, provoking controversy both as to the roles of astrocytic GPCRs and the utility of DREADDs for investigating their activity. In this review we question how accurately DREADDs can replicate endogenous GPCR stimulation in adult mouse hippocampal astrocytes, and, by extension, how the use of DREADDs in hippocampal astrocytes may inform us on the roles astrocytic GPCRs in cognitive processes such as learning and memory.

## 2. Astrocyte participation at the synapse

### 2.1. Astrocyte morphological and functional responses to synaptic activity

**FIGURE 1:**
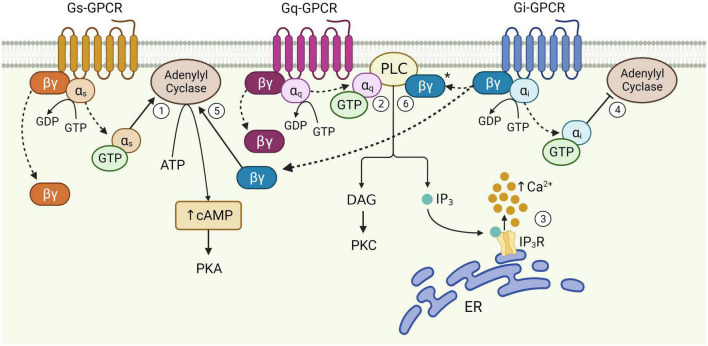
Schematic of astrocytic G-protein coupled receptor (GPCR) signaling. Activation of membrane-bound astrocytic GPCRs triggers the exchange of GDP for GTP at the α subunit and dissociation of α and βγ subunits from the receptor (dotted arrows). Once released, α_s_ stimulates adenylyl cyclase activity leading to the production of cAMP from ATP (1). α_q_ binds PLC, catalyzing diacylglycerol (DAG) and IP_3_ synthesis (2). IP_3_ binds its receptor on the endoplasmic reticulum (ER) to release Ca^2+^ from internal stores (3). α_i_ inhibits adenylyl cyclase activity (4). The Gi βγ subunit can potentiate cAMP elevations mediated by Gs-GPCRs (5), and is speculated to enhance Gq-GPCR-mediated Ca^2+^ release from stores *via* the PLC-IP_3_ pathway (6), although this has yet to be proven in astrocytes (*). Figure created with Biorender.com.

Astrocytes, originally named for their “star”-like appearance, extend thousands of highly ramified processes across the neuropil, with a single mouse astrocyte capable of contacting 300–600 dendrites and over 100,000 synapses (Halassa et al., 2007). The abundance of ultrathin PAPs across the entire cell give astrocytes a sponge-like morphology (Arizono et al., 2020) that mediates interactions with neurons across largely non-overlapping spatial domains of interconnected astrocytes (Bushong et al., 2002). The nanoscopic scale of PAPs and their close physical proximity to the synaptic cleft makes them perfectly situated to mediate bidirectional communication with neurons (Ghézali et al., 2016), and by extension, survey and influence the activity of a well-defined volume of the neuropil (Papouin et al., 2017). Astrocytes express a wide range of cell surface receptors, including GPCRs (Verkhratsky, 2009), some of which allow for the transduction of neuronal signals into intracellular Ca^2+^ and cAMP elevations when stimulated by neurotransmitters (such as glutamate and ATP) released by neurons (Zhou et al., 2019; Park and Lee, 2020; Kofuji and Araque, 2021). Astrocytic cAMP levels are regulated primarily by activation of different astrocytic GPCRs that can stimulate or inhibit the generation of cAMP from ATP by action of adenylyl cyclase activity (Figure 1; Zhou et al., 2019). Astrocytic cAMP levels can modulate morphological and functional characteristics of these cells. For example, elevated intracellular cAMP by activation of beta-adrenoceptors (βARs) is known to influence morphology of cultured astrocytes (Vardjan et al., 2014). Functionally, astrocytic cAMP is known to trigger a number of signal transduction pathways leading to glycogenolysis, glucose uptake and lactate release, all of which have proposed functional roles in mediating gliotransmission and plasticity mechanisms (Zhou et al., 2019, 2021). Indeed, cAMP elevations during memory formation are known to induce synaptic plasticity mechanisms in the adult mouse hippocampus, resulting in improved memory acquisition (Zhou et al., 2021). The cAMP pathway is also known to regulate the neuroinflammatory profile of astrocytes, whereby increased intracellular cAMP levels protects against proinflammatory insults by impairing upregulation of pro-inflammatory cytokines (Christiansen et al., 2011; Zhou et al., 2019; Kim et al., 2021).

While cAMP levels seem to impact astrocytic GPCR-related synaptic and behavioral effects, much more is known about the effects of GPCRs on astrocytic Ca^2+^ signaling. Activation of GPCRs allow astrocytes to produce activity-dependent Ca^2+^ signaling in response to synaptic activity, which is thought to be a key mediator of astrocyte-neuron interactions, including mechanisms that influence synaptic transmission (Perea and Araque, 2005; Di Castro et al., 2011) and plasticity (Guerra-Gomes et al., 2018; Liu J.-H. et al., 2022). A major source of astrocytic Ca^2+^ is that released from the endoplasmic reticulum, which is mediated primarily by activation of intracellular inositol trisphosphate (IP_3_) receptors *via* the phospholipase C (PLC) signaling pathway (Figure 1; Volterra and Meldolesi, 2005; Agulhon et al., 2008). It has recently been shown that endoplasmic reticulum can be found locally in PAPs of the mouse cortex (Aboufares El Alaoui et al., 2021), suggesting that PLC-IP_3_-dependent Ca^2+^ elevations can occur locally within PAPs, without necessarily involving the cell soma. Changes to intracellular Ca^2+^ levels are therefore dependent upon diverse inputs received at receptors and the intracellular signaling pathways that mediate Ca^2+^ elevations – both of which are subject to intrinsic properties of the receptor, astrocyte and the neural circuit to which they belong (Durkee and Araque, 2019).

Accordingly, compartmentalized Ca^2+^ events at PAP microdomains appear to be much more prevalent than global Ca^2+^ signaling involving the soma and proximal processes (Shigetomi et al., 2013; Haustein et al., 2014; Srinivasan et al., 2015; Rungta et al., 2016; Bindocci et al., 2017; Lia et al., 2021; Sherwood et al., 2021). Indeed, Ca^2+^ events within astrocytic somata likely originate from the fine processes (Oe et al., 2020). The basal distribution patterns of intracellular Ca^2+^ are therefore heterogenous both within and between cells (Zheng et al., 2015) whereby these basal concentrations appear to be functionally relevant to evoked Ca^2+^ signaling (King et al., 2020). That is, resting state Ca^2+^ concentrations in PAPs are correlated with the amplitudes of evoked Ca^2+^ elevations in the same PAPs, inviting the possibility that PAPs can encode information regarding the physiological demands of the surrounding neuropil. Astrocyte morphology is also shown to be highly heterogenous across different brain regions, according to the functional demands of nearby circuits (Chai et al., 2017; Matias et al., 2019). For example, while hippocampal and striatal astrocytes in the adult mouse both show equivalent somatic volumes and number of primary branches, striatal astrocytes occupy larger spatial territories, while hippocampal astrocytes contact more excitatory synapses and have PAPs positioned much closer to the post-synaptic densities than their striatal counterparts (Chai et al., 2017).

Not only do PAPs exhibit the ability to sense and adapt to the functional demands of their surroundings, morphological plasticity of PAPs is also associated with synaptic remodeling, with functional consequences to memory (Badia-Soteras et al., 2022; Lawal et al., 2022). β-adrenoceptors have been shown to affect the morphology of astrocyte *in vitro* (Vardjan et al., 2014), while metabotropic glutamate receptor 3 (mGluR3) and 5 (mGluR5) and IP3R2-mediated calcium release are involved in PAP plasticity (Lavialle et al., 2011; Perez-Alvarez et al., 2014). Accordingly, stimulation of PLC-IP_3_-dependent Ca^2+^ signaling, a key effect of astrocytic GPCR activation, has been shown to mediate structural remodeling of PAP-neuronal contacts *in vivo* (Tanaka et al., 2013; Bernardinelli et al., 2014; Perez-Alvarez et al., 2014). Indeed, PAP motility is also shown to be functionally relevant to long-term potentiation (LTP) whereby PAPs transiently alter their coverage of postsynaptic spines during LTP induction (Bernardinelli et al., 2014; Santello et al., 2019; Henneberger et al., 2020). This suggests that PAP-spine contacts are selectively restructured in an activity-dependent manner as a function of LTP. Given synaptic remodeling is associated with learning and memory (Caroni et al., 2012; Badia-Soteras et al., 2022), and morphological plasticity of PAPs was found to be impaired in a mouse model of Alzheimer Disease (Kater et al., 2023), astrocytic GPCR-mediated PAP motility is likely a key mechanism mediating learning and memory processes.

### 2.2. GPCRs and associated signaling pathways

The functional responses of astrocytes to synaptic activity described above are evoked primarily through activation of cell surface GPCRs. GPCRs are a large group of membrane-bound, metabotropic receptors, whose activation stimulates several cellular signaling pathways (Rosenbaum et al., 2009). They are characterized by seven transmembrane α-helix domains, the intracellular portions of which interact with heterotrimeric G proteins, composed of α, β, and γ subunits (Wettschureck and Offermanns, 2005). In the resting, inactive state, the α-subunit is bound to guanosine diphosphate (GDP) and remains associated with the βγ complex. Activation of the GPCR triggers exchange of GDP for guanosine triphosphate (GTP) on the α-subunit, resulting in its dissociation from the βγ-dimer and the receptor (Figure 1). This dissociation allows for the Gα subunit and βγ dimer to separately interact with other membrane proteins, such as adenylyl cyclase and PLC, and stimulate downstream signaling pathways (Weis and Kobilka, 2018). The structural nature of this system allows for substantial functional versatility. That is, all three components; receptor, G-protein and downstream effectors, are subject to regulation by additional mechanisms, while interactions between each component may also introduce variation, allowing GPCRs to evoke a wide variety of functions through stimulation of signaling pathways in multiple cell types – including astrocytes (Wettschureck and Offermanns, 2005).

G-protein coupled receptors are often categorized as Gq, Gs, or Gi, according to structural and functional similarities of the heterotrimeric G-protein α-subunit with which they preferentially interact (Syrovatkina et al., 2016). However, the effects of GPCR activity are made significantly more complex given that the same GPCR can couple to multiple Gα isoforms with different affinities, according to their specific receptor conformations (Rosenbaum et al., 2009; Okashah et al., 2019; Sandhu et al., 2019). This binding promiscuity allows for the same receptor to stimulate multiple signaling pathways to different extents. Nevertheless, the downstream effects of each GPCR subtype vary according to the major signaling pathways they preferentially activate (Figure 1). Gi-GPCR stimulation typically inhibits cAMP production by adenylyl cyclase inhibition. This has an inhibitory effect on neurons, whereby downregulation of cAMP and PKA mediate membrane hyperpolarization by increasing the open probability of G-protein inward rectifier K^+^ channels (Armbruster et al., 2007; Witkowski et al., 2012; Durkee et al., 2019). However, Gi-GPCR stimulation in astrocytes can elicit Ca^2+^ elevations, a marker of cellular activation, alongside reduced (Sobolczyk and Boczek, 2022) or elevated cAMP levels (Tang and Gilman, 1991; Walker et al., 2017). Conversely, Gs-GPCRs upregulate cAMP production and are therefore regarded as stimulatory in neurons – however are generally reported to not mediate Ca^2+^ elevations in astrocytes (Shigetomi et al., 2019; Oe et al., 2020). Finally, astrocytic Gq-GPCRs stimulate the PLC signaling pathway, mediating IP_3_ production and subsequent Ca^2+^ release from the endoplasmic reticulum (Rhee and Bae, 1997; Volterra and Meldolesi, 2005; Agulhon et al., 2008).

### 2.3. Investigating the effects of astrocytic GPCRs on synapse and behavior

A variety of GPCRs are endogenously expressed on hippocampal astrocytes (Verkhratsky et al., 2019), and studying their activation effects provides insight into key functional characteristics of astrocytes, including their effects on neuronal activity. Astrocytes exhibit substantial morphological and functional heterogeneity across different brain regions (Matias et al., 2019; Batiuk et al., 2020), species (Falcone, 2022) and developmental time (Sun et al., 2013; Farhy-Tselnicker and Allen, 2018; Matias et al., 2019). As a result, understanding the functional roles of astrocytic GPCRs requires careful consideration of the species, brain region and developmental timepoints under investigation. To limit confounds due to astrocyte functional heterogeneity and GPCR expression, here we compare literature on DREADDs and endogenous GPCRs primarily in adult mouse hippocampal astrocytes.

A major limitation to our understanding of the role of astrocytic GPCRs in synaptic activity and memory has been the methodological challenge of targeting these receptors to specific cell types within the intact brain. Classical pharmacological studies modulating astrocytic GPCR activity by agonist application (Angulo et al., 2004; Fiacco and McCarthy, 2004; Rae and Irving, 2004) are criticized for being unable to specify these interventions to astrocytes without impacting activity of the same receptors present on nearby neurons and other glia. Hence, although these studies indicate a role for astrocytic GPCRs in synaptic activity, their limitations hamper the drawing of proper conclusions. Selective elevation of Ca^2+^ by photochemical uncaging in astrocytes circumvented these issues, and was shown by multiple groups to have impacts on neuronal function in hippocampal slice preparations (Fellin et al., 2004; Liu et al., 2004). However, this technique lacks physiological plausibility, given that Ca^2+^ elevations are evoked in somata, failing to replicate endogenous spatiotemporal dynamics of Ca^2+^ signaling in PAPs (Shigetomi et al., 2013; Bindocci et al., 2017; Lia et al., 2021). Furthermore, because the uncaging of Ca^2+^ bypasses GPCR activation and subsequent signaling cascades, the complexity of this mechanism is lost and resulting Ca^2+^ dynamics are likely incomparable to those elicited through receptor activation.

## 3. DREADDs

### 3.1. Next generation methodology

The advent of transgenic mouse models and chemogenetic tools has enabled the selective activation of GPCRs in astrocytes. The first of these systems utilized the Mas-related genes A1 receptor (MrgA1R), a GPCR endogenously expressed in nociceptive sensory neurons in the dorsal root and trigeminal ganglia, but not in the brain (Dong et al., 2001). Chemogenetic expression of MrgA1R in hippocampal astrocytes thereby allowed for selective activation of Gq-GPCR-mediated Ca^2+^ signaling by exogenous ligand application (Fiacco et al., 2007; Agulhon et al., 2010). However, since agonists for MrgA1R cross the blood brain barrier with very poor efficacy, *in vivo* applications of this technique remained somewhat out of reach (Xie et al., 2015). Very soon after, a new family of engineered GPCRs – DREADDs – were developed, making *in vivo* modulation of GPCR signaling specifically in astrocytes a reality (Armbruster et al., 2007). DREADDs were engineered through molecular evolution of human muscarinic cholinergic receptors. The Gq-DREADD hM3Dq, and the Gi-DREADD hM4Di, were derived from the human M3 and M4 receptors respectively, while the Gs-DREADD rM3Ds was derived from the rat M3 receptor, mutated to contain the 2nd and 3rd intracellular loop of the turkey beta adrenoceptor (βAR) to allow for interaction with Gs proteins (Armbruster et al., 2007; Guettier et al., 2009; Farrell et al., 2013; Shen et al., 2021). Endogenous GPCRs show binding promiscuity between subtypes whereby receptor binding to each G protein subtype is reflected by conformational changes to the third intracellular loop of the GPCR structure (Sandhu et al., 2019). DREADDs on the other hand, are often assumed to activate generalized downstream signaling mechanisms specific to Gq-, Gs-, and Gi-GPCRs. However, since DREADD-mediated effects on Gq-, Gs-, and Gi-signaling are based upon the structure of their parent receptor (M3, M3/β_1_AR or M4), it is likely that the specificity of their G-protein coupling mechanisms reflect those of M3, M4 and β_1_A receptors, which have been shown to also couple multiple G-protein subtypes, albeit with lower affinities to their preferentially bound Gα subunit (Rubenstein et al., 1991; Dittman et al., 1994; Ilyaskina et al., 2018). As a result, researchers should consider that DREADDs do not interact specifically with Gq-, Gs-, and Gi-protein subtypes, but rather can couple multiple subtypes with varied affinities, much like their endogenous parent receptors.

Designer Receptors Exclusively Activated by Designer Drugs can be incorporated into cells of interest in living animals by viral expression using a cell-specific promotor [e.g., for astrocytic expression, using GFAP promotors (Chai et al., 2017; Adamsky et al., 2018; Durkee et al., 2019; Nagai et al., 2019; Lei et al., 2022)], and activated by systemic ligand injection (Adamsky et al., 2018; Atasoy and Sternson, 2018). The DREADD technique was first applied to study astrocytic GPCR signaling *in vivo* by Agulhon et al. (2013), with a number of major advantages. First, it allows for the modulation of activity in specific cell populations, whilst also retaining the complexity of GPCR-mediated signaling cascades (Xie et al., 2015; Atasoy and Sternson, 2018). This method is also relatively non-invasive compared to other *in vivo* experimental systems such as optogenetics. This is because activation of the transfected DREADD can be achieved by intraperitoneal ligand injection, while optogenetics requires the application of light directly to brain tissue (Xie et al., 2015). Second, the development of DREADDs mimicking different GPCR subtypes increased the number of experimental applications of this method compared to Gq-coupled MrgA1R (Fiacco et al., 2007; Atasoy and Sternson, 2018). Finally, unlike MrgA1R ligands, CNO ligands can cross the blood brain barrier very efficiently (Bender et al., 1994), allowing for *in vivo* DREADD activation and the assessment of subsequent behavioral effects (Xie et al., 2015).

### 3.2. Experimental findings from DREADD studies in astrocytes

Despite boasting increased physiological plausibility compared to other methods, recent reports highlight variable effects upon DREADD activation in astrocytes, including effects on Ca^2+^ dynamics, synaptic activity (e.g., Chai et al., 2017; Durkee et al., 2019) and behavior (e.g., Nam et al., 2019; Kol et al., 2020), even when the same DREADD is used in the same brain region, species and during the same developmental period. This evokes important questions yet to be assessed: Why do results from studies using DREADDs vary? Is this variability relevant to our understanding of endogenous GPCRs, or must we consider in greater depth the limitations of DREADDs? Here we question the assumption that astrocytic DREADD stimulation accurately mimics endogenous GPCR activity in astrocytes and subsequent synaptic and behavioral effects. To do this, we will first discuss the known intracellular, synaptic and behavioral effects of experimental activation of astrocytic Gq, Gs-, and Gi-DREADDs in the adult mouse hippocampus.

#### 3.2.1. Gq-DREADDs

Gq-DREADDs such as hM3Dq have been experimentally expressed in astrocytes to replicate Gq-GPCR activation and investigate the subsequent intracellular, synaptic and behavioral effects *in vivo* (Roth, 2016). Stimulation of Gq-DREADDs has been shown to elicit Ca^2+^ signaling in astrocytic somata and processes in the mouse hippocampus, *via* IP_3_-dependent mechanisms (Agulhon et al., 2013; Chai et al., 2017; Adamsky et al., 2018; Durkee et al., 2019; Van Den Herrewegen et al., 2021; Liu J.-H. et al., 2022). Interestingly, Liu J.-H. et al. (2022) showed that while the majority of evoked somatic Ca^2+^ elevations are IP_3_ receptor 2 (IP_3_R2)-dependent, those in processes may be largely IP_3_R2-independent. Gq-DREADD-induced Ca^2+^ events are relatively long lasting compared to Ca^2+^ events evoked by Gi-DREADD stimulation (Kim et al., 2021). However, evoked and spontaneous Ca^2+^ events are shown to be completely abolished after initial elevations upon CNO injection (Vaidyanathan et al., 2021) despite CNO remaining available for binding for much longer (about 2 h). Together, these studies indicate that while Gq-DREADD ligand binding increases Ca^2+^ signaling *in vivo* by IP_3_R2-dependent and -independent mechanisms, Ca^2+^ activity is silenced upon prolonged stimulation.

While it is clear that astrocytic Gq-DREADD activations evoke intracellular Ca^2+^ elevations, the impact of these Ca^2+^ events to synaptic activity has become a point of controversy (Bazargani and Attwell, 2016). A number of studies using the MrgA1R chemogenetic tool in adult mouse hippocampal astrocytes have shown that although Gq pathway stimulation evokes robust Ca^2+^ elevations, it has no effect on neuronal activity *in situ* (Fiacco et al., 2007; Agulhon et al., 2010). However, since then, other studies have shown that Gq-DREADD activation in adult mouse hippocampal astrocytes can impact synaptic activity through interactions with nearby neurons. It was shown by Adamsky et al. (2018) that astrocytic Gq-DREADD stimulation increases evoked miniature excitatory post-synaptic currents (mEPSCs) by approximately 50%. Furthermore, Durkee et al. (2019) showed that Gq-DREADD activation in astrocytes robustly increases the frequency of slow inward currents (SICs) in neighboring neurons. This finding opposes that from Chai et al. (2017) who did not detect changes to SICs upon Gq-DREADD activation in astrocytes. However, Gq-DREADD-induced astrocytic activity in CA1 has been shown to be necessary and sufficient to induce NMDA-dependent LTP in CA3-CA1 synapses and enhance memory processes including acquisition and early consolidation (Adamsky et al., 2018). SICs are known to mediate NMDAR activity and plasticity mechanisms (Papouin and Oliet, 2014), substantiating claims that Gq-DREADD activation on hippocampal astrocytes alters synaptic activity. Indeed, more recent studies have also shown that Gq-DREADD stimulation on CA1 astrocytes mediates long-lasting potentiation at synapses (Van Den Herrewegen et al., 2021; Liu J.-H. et al., 2022).

These Gq-DREADD-mediated synaptic effects are also shown to influence memory. Adamsky et al. (2018) show that astrocytic Gq-DREADD activation in mouse CA1 selectively increased neuronal activity during fear memory acquisition, leading to memory enhancement. Furthermore, blocking astrocytic Gq-DREADD-induced Ca^2+^ elevations in the hippocampus using IP_3_R2 knockout mice evoked significant impairments to LTP and remote fear memory compared to wild type (Liu J.-H. et al., 2022).

Together, it seems that various *in situ* and *in vivo* studies corroborate the view that Gq-DREADD activation in astrocytes and subsequent Ca^2+^ signaling can (i) impact synaptic activity by generation of mEPSCs, SICs and LTP, and (ii) plays a role in memory formation. Indeed, as research in this area progresses, astrocytic Gq-DREADD activation and the synaptic and behavioral consequences are becoming increasingly established. However, the specific mechanisms by which astrocytes exert these effects, downstream of Ca^2+^ elevations, remains a debated topic for future research.

#### 3.2.2. Gs-DREADDs

A limited number of studies have examined the activity of Gs-DREADD activation in astrocytes. Oe et al. (2020) showed that astrocytic Gs-DREADD activation in the adult mouse hippocampus mediates sustained elevation of cAMP levels and subsequently stimulates glycogenolysis. In addition, Gs-DREADD activation in hippocampal astrocytes elicits Ca^2+^ elevations (Chai et al., 2017; Nagai et al., 2021). Other studies have used alternative chemogenetic means to probe Gs-GPCR activity, including use of receptors activated solely by synthetic ligands (RASSL) such as RASSL serotonin 1 (Rs1), a Gs-coupled receptor based on the human 5-HT4b receptor. In one such study, stimulation of Rs1 in hippocampal astrocytes of adult mice was seen to increase cAMP levels, with no effect on Ca^2+^ signaling, and was shown to impair memory consolidation, but not learning (Orr et al., 2015). Together, these studies confirm that Gs-DREADDs expressed in the hippocampus of adult mice evoke increased cAMP levels and subsequent memory impairment. However, while Gs-DREADDs can indeed elicit Ca^2+^ elevations, this is not always the case. The cause of this inconsistent Ca^2+^ response to Gs-DREADD stimulation is unknown, and further research is required to elucidate the mechanisms responsible.

#### 3.2.3. Gi-DREADDs

Several studies have shown that Gi-DREADD activation in mouse hippocampal astrocytes elicits Ca^2+^ elevations (Durkee et al., 2019; Kol et al., 2020; Kim et al., 2021), while others show limited to no effects on astrocytic Ca^2+^ levels (Chai et al., 2017; Van Den Herrewegen et al., 2021), despite all studies utilizing the same Gi-DREADD construct (hM4Di). It was shown by two studies that Gi-DREADD-evoked Ca^2+^ elevations are IP_3_R2-dependent (Kol et al., 2020; Vaidyanathan et al., 2021), indicating that Gi-DREADD activation evokes Ca^2+^ release from intracellular stores. Furthermore, when present, Gi-DREADD-evoked Ca^2+^ activity in hippocampal astrocytes is distinct to that of Gq-DREADDs, whereby Gi-mediated Ca^2+^ fluctuations are more transient in nature (Kim et al., 2021). Indeed, Kol et al. (2020) showed that Gi-DREADD activation in CA1 astrocytes evokes a moderate decrease in astrocytic Ca^2+^ levels with respect to baseline, after an initial transient peak in activity. These Ca^2+^ fluctuations also seem to regulate the reactive, proinflammatory astrocyte phenotype, such that prolonged Gi-DREADD activation results in inhibition of Ca^2+^ transients triggered by proinflammatory environments, and is associated with ameliorating neuroinflammation and associated cognitive impairment (Kim et al., 2021).

Gi-DREADD-evoked increases in intracellular Ca^2+^ levels in adult mouse hippocampal astrocytes is accompanied by profound effects on nearby neurons, including increased neuronal firing rates and *SIC* frequency (Durkee et al., 2019). Activation of astrocytic Gi-DREADDs in the adult mouse during learning has also been shown to potentiate NMDAR-dependent synaptic plasticity in Schaffer collaterals by lowering the stimulation threshold for LTP, ultimately resulting in enhanced recent contextual memory (Nam et al., 2019). Astrocytic Gi-DREADD-mediated effects on synaptic activity in Schaffer collaterals has also been shown in the absence of evoked Ca^2+^ signaling, whereby Gi-DREADD activation increased field excitatory post-synaptic potential (fEPSP) amplitude and LTP at CA1 synapses in adult mice (Van Den Herrewegen et al., 2021). However, in contrast, behavioral results from Kol et al. (2020) indicate that Gi-DREADD activation in CA1 astrocytes of adult mice during learning has no effect on recent memory retrieval, but does disrupt remote memory retrieval. It is noted that no effects on local CA1 neuronal activity were observed, but rather impaired remote memory retrieval resulted from impaired recruitment of CA1 projections during acquisition and disrupted synaptic transmission from the hippocampus to the cortex (Kol et al., 2020). This stark contrast between the results of Nam et al. (2019) and Kol et al. (2020) could be due to differences in (i) cellular pathways evoked by Gi-DREADD stimulation (Gα or Gβγ subunit affecting Ca^2+^ or cAMP), (ii) type of hippocampal synapse targeted (CA3-CA1 vs. CA1-cortex) or (iii) experimental design, whereby the potentiation shown by Nam et al. (2019) and Van Den Herrewegen et al. (2021) was dependent upon electrical stimulation of nearby neurons, while Kol et al. (2020) did not provide this stimulation.

Relatively little is known about Gi-DREADD effects on cAMP activity. However, what has been shown is that Gi-DREADD activation in rat hippocampal astrocytes inhibits cAMP production and attenuates stress-enhanced fear learning (Jones et al., 2018). Further studies are required to confirm that this also occurs in the adult mouse hippocampus.

Together, these studies show that Gi-DREADD activation can evoke at least two effector pathways – one that is Ca^2+^-dependent and another, Ca^2+^-independent pathway, likely relying on cAMP inhibition to evoke downstream effects. In addition, these studies show, similarly to Gs-DREADDs, that astrocytic Gi-DREADD stimulation can evoke Ca^2+^ signaling in many, but not all cases, sparking controversy on the mechanism of action of Gi-DREADD activation in astrocytes. However, the finding that Gi-DREADD activation in adult mouse hippocampal astrocytes evokes synaptic changes by both Ca^2+^-dependent and -independent mechanisms, illustrates the complexity of astrocytic GPCR functionality with respect to intracellular Ca^2+^ dynamics and resulting synaptic effects. Despite reduced cAMP production being a hallmark of this GPCR subtype, very little is known about the effects of Gi-DREADD stimulation on cAMP levels in hippocampal astrocytes. However, we speculate that reduced cAMP levels in the mouse hippocampus mediate Ca^2+^-independent synaptic and behavioral effects of astrocytic Gi-DREADD stimulation, whereby the type and frequency of stimulation determines downstream effects.

Taken together, the current understanding of the intracellular, synaptic and behavioral effects of DREADD expression in adult mouse hippocampal astrocytes is reaching a loose consensus. What many of these DREADD studies in astrocytes have made clear, is that much more research is needed to unravel the exceptional complexity of astrocytic GPCR systems and fully characterize their downstream effects on synapses and behavior. To understand these mechanisms, it is therefore important to evaluate what is known about endogenous GPCR signaling in astrocytes, and determine to what extent the effects of DREADD and endogenous GPCR stimulation are comparable in astrocytes.

## 4. Endogenous astrocytic GPCRs

### 4.1. Endogenous GPCR expression in astrocytes

Endogenously expressed GPCRs can be categorized as Gq, Gs, and Gi, thereby corresponding to the DREADD subtypes previously described. Here we provide evidence for the presence of some of the most frequently studied GPCRs in the adult mouse hippocampus (summarized in Tables 1–3) and describe the known intracellular, synaptic and behavioral effects of their activation, such that we may later compare these effects to those of DREADD stimulation in section “5. Comparative assessment of endogenous GPCRs and DREADDs.”

**TABLE 1:** Endogenous Gq-GPCRs in adult mouse hippocampus: Synaptic and behavioral effects.

GPCR	Agonists	Astrocyte response	Synaptic response	Hippocampus-related behavior	References
mGluR1	Glutamate *DHPG* *t-ACPD*	↑ Ca^2+^ by PLC-IP_3_ signaling	Generation of SICs, LTP induction		* Fellin et al., 2004; * Perea and Araque, 2005; Chai et al., 2017
mGluR5	Glutamate *DHPG* *t-ACPD* *CHPG*	↑ Ca^2+^ by PLC-IP_3_ signaling	Generation of SICs, LTP induction, PAP plasticity, mEPSCs		* Fellin et al., 2004; * Perea and Araque, 2005; * Lavialle et al., 2011; Liu C. et al., 2022
P2YR1	ATP *2MeSADP* *MRS2365*	↑ Ca^2+^ by PLC-IP_3_ signaling	NMDAR-dependent synaptic potentiation	AD-related memory impairment	Domercq et al., 2006; Tang et al., 2015; Reichenbach et al., 2018; Shigetomi et al., 2018
α1AR	Phenylephrine	↑ Ca^2+^ by PLC-IP_3_ signaling	Regulation of inhibitory neurotransmission		Srinivasan et al., 2015; Chai et al., 2017; Batiuk et al., 2020
CB1R	2-AG Anandamide *Δ9-THC* *WIN55,212-2*	↑ Ca^2+^ by PLC-IP_3_ signaling Gi-mediated ↓ cAMP levels	NMDAR-dependent SICs, LTP and LTD induction	Memory extinction	Marsicano et al., 2002; Navarrete and Araque, 2010; Han et al., 2012; Navarrete et al., 2014; Gómez-Gonzalo et al., 2015; Gutiérrez-Rodríguez et al., 2018

* = rat; pharmacological agonists = *italics*.

**TABLE 2:** Endogenous Gs-GPCRs in adult mouse hippocampus: Synaptic and behavioral effects.

GPCR	Agonists	Astrocyte response	Synaptic response	Hippocampus-related behavior	References
A2AR	Adenosine CGS 21690	↑ cAMP	Neuroinflammation Inhibits NO production	↓ Memory consolidation	Brodie et al., 1998; Boison et al., 2010; Orr et al., 2015; Shigetomi et al., 2019; Zhou et al., 2019; Batiuk et al., 2020
βARs	Noradrenaline *Zinterol Isoprenaline*	↑ cAMP Lactate release (β_2_AR only)	LTP induction	↑ Memory consolidation	* Gao et al., 2016; Oe et al., 2020

* = rat; pharmacological agonists = *italics*.

**TABLE 3:** Endogenous Gi-GPCRs in adult mouse hippocampus: Synaptic and behavioral effects.

GPCR	Agonist	Astrocyte response	Synaptic response	Hippocampus-related behavior	References
CB1R	2-AG Anandamide *Δ9-THC*	↑ Ca^2+^ by PLC-IP_3_ signaling Gi-mediated ↓ cAMP levels	NMDAR-dependent SICs, LTP and LTD induction	Memory extinction?	Marsicano et al., 2002; Navarrete and Araque, 2008, 2010; Han et al., 2012; Gómez-Gonzalo et al., 2015; Gutiérrez-Rodríguez et al., 2018
mGluR3	Glutamate *NAAG* *LY379268* *LY354740*	↓ cAMP, βγ-mediated ↑ cAMP - βAR potentiation ↑ Ca^2+^ by PLC-IP_3_ signaling	Release of neurotrophic factors, LTP induction	Memory extinction?	Sun et al., 2013; Haustein et al., 2014; Chai et al., 2017; Walker et al., 2017
GABAbR	GABA *R-baclofen*	* ↓ cAMP levels ↑ Ca^2+^ by PLC-IP_3_ signaling	LTP, LTD, reduced fEPSPs		* Patte et al., 1999; Haustein et al., 2014; Chai et al., 2017; Covelo and Araque, 2018; Durkee et al., 2019; Batiuk et al., 2020

* = cultured rat astrocytes; pharmacological agonists = *Italics;* ? = unconfirmed.

#### 4.1.1. Endogenous Gq-GPCRs

Metabotropic glutamate receptors (mGuRs) are a core group of GPCRs present in hippocampal astrocytes of the adult mouse. Multiple mGluR subtypes exist, two of which are Gq-coupled; mGluR1 and mGluR5, whose activation elicits IP_3_-dependent Ca^2+^ signaling as previously described (Table 1). mGluR1 and mGluR5 are both group I mGluRs (mGluR1/5) and are therefore studied within this grouping, as well as individually. Substantial evidence shows the presence of mGluR1 (Fellin et al., 2004; Xie et al., 2012; Devaraju et al., 2013; Chai et al., 2017; Batiuk et al., 2020) and mGluR5 (Fellin et al., 2004; Chai et al., 2017; Batiuk et al., 2020; Liu C. et al., 2022) in rodent hippocampal astrocytes *in vitro* and *in situ*. However, relatively few studies have illustrated mGluR1/5 expression specifically in hippocampal astrocytes in the adult mouse (Chai et al., 2017; Batiuk et al., 2020; Liu C. et al., 2022). Immunoelectron microscopy by Sun et al. (2013) provided strong evidence that mGluR5 expression is downregulated over developmental time to near absence in the adult mouse brain. Indeed, although many studies have detected mGluR1/5 expression in rodent hippocampal astrocytes, single cell RNA sequencing (scRNA-seq) data also shows that expression tends to be very limited in the adult mouse hippocampus (Chai et al., 2017; Batiuk et al., 2020), indicating that mGluR5 is more relevant to developmental processes than those in the mature mouse. However, expression of astrocytic mGluR5 has since been shown by immunohistochemistry in the adult mouse hippocampus (Liu C. et al., 2022). Expression of astrocytic mGluR5 is also shown in the adult rat hippocampus, whereby expression is localized predominantly in PAPs (Lavialle et al., 2011). Here, Lavialle et al. (2011) also implicate PAP mGluR5 expression in PAP motility indicating that (i) expression levels may be higher in PAPs compared to somata, and (ii) astrocytic mGluR5 is functionally significant to synaptic activity through effects on PAP motility. It is possible that high degrees of heterogeneity in astrocytic gene expression impacts detection of GPCRs in whole cell analysis and minimizes potential contributions of mGluR5 to astrocyte-synapse interactions. Functionally, mGluR1/5s are also shown to exhibit dose-dependent response heterogeneity to agonist application, whereby low concentrations of agonists elicit single peak signals, and progressively higher concentrations produce multi-peak and plateaued responses in hippocampal astrocytes of young mice (Xie et al., 2012); differences which are illustrative of how Ca^2+^ dynamics vary according to GPCR receptor stimulation frequency and duration (Tang et al., 2015). However, the downstream implications of these differences in evoked Ca^2+^ responses are not fully understood and require further investigation, particularly in the adult mouse. Astrocytic mGluR1/5 activation and subsequent Ca^2+^ activity is also required for the generation of NMDAR-dependent SICs in neurons, thereby facilitating LTP in the rodent hippocampus (Fellin et al., 2004; Perea and Araque, 2005). Downregulated expression of astrocytic mGluR5 in the adult mouse CA1 has been shown to reduce the frequency of miniature excitatory post-synaptic currents (mEPSCs), thereby impairing excitatory synaptic function in this region (Liu C. et al., 2022). Furthermore, mGluR5 overexpression rescued these impairments. Although the mechanism by which this occurs is debated, it may involve increased astrocytic Ca^2+^ elevations and probability of gliotransmitter release. Accordingly, mGluR1/5 are frequently cited as likely mediators of astrocytic glutamate exocytosis and gliotransmission (Volterra and Meldolesi, 2005; Hamilton and Attwell, 2010; Mahmoud et al., 2019).

The P2Y purinoceptor 1 (P2YR1) is another Gq-coupled GPCR shown to be present in PAPs located at excitatory synapses in mouse hippocampal slices (Domercq et al., 2006; Santello et al., 2011; Shigetomi et al., 2018). It has been shown that P2YR1-PLC-IP_3_- mediated Ca^2+^ elevations induce gliotransmission and NMDAR-dependent synaptic potentiation. However, these synaptic effects of astrocytic P2YR1-mediated Ca^2+^ elevations are not sufficient on their own but require the presence of permissive, homeostatic factors like TNFα (Domercq et al., 2006; Santello et al., 2011). Furthermore, while mGluR1 antagonist application reduced Ca^2+^ signaling in PAPs, combined application of both mGluR1 and P2YR1 antagonists reduced Ca^2+^ signaling even further (Tang et al., 2015). Hence, it is likely that both receptors contribute to Gq-mediated synaptic effects at mouse CA3-CA1 synapses in accordance with surrounding neuronal activity (Tang et al., 2015) and the presence of supporting proteins such as TNFα, controlling stimulus-secretion coupling in astrocytes (Santello et al., 2011). However, while the above evidence indicates that endogenous Gq-GPCR activation in astrocytes promotes intracellular and synaptic processes that are expected to enhance memory, Reichenbach et al. (2018) show that inhibition of astrocytic P2YR1 is protective against spatial learning and memory impairment in the APP/PS1 mouse model of Alzheimer’s Disease (AD). In this case, the maladaptive impact of astrocytic P2YR1 on memory is likely due to the pathology of this AD model (including P2YR1 hyperactivity and gliosis), as opposed to being a reflection of the role of astrocytic P2YR1 in memory under physiological conditions – which remains to be determined.

Cannabinoid receptor 1 (CB1R) is a GPCR that depending upon agonist availability, can couple to either Gi or Gq proteins (Lauckner et al., 2005), but preferentially to Gi proteins, and will therefore be discussed in more detail below (section “4.1.3. Endogenous Gi-GPCRs”).

The presence of the Gq-coupled α1-adrenoceptor (α1AR) on adult mouse hippocampal astrocytes has been illustrated *in vivo* (Srinivasan et al., 2015), *in situ* (Chai et al., 2017) and through scRNA-seq (Batiuk et al., 2020). This receptor seems to be necessary for the initial peak in startle-evoked intracellular Ca^2+^ activity, but not for slow rising elevations thereafter (Srinivasan et al., 2015). This finding is substantiated by those of Oe et al. (2020) who show α1AR stimulation in cortical astrocytes mediates sharp elevations in intracellular Ca^2+^ upon short stimulus durations, and surges of Ca^2+^ activity upon neuronal burst firing. While this was observed in cortical regions, we speculate that this mechanism also holds true in the hippocampus given the findings from Srinivasan et al. (2015), who showed that α1AR-mediated Ca^2+^ elevations display peaks of activity as opposed to prolonged elevations. Astrocytic α1AR are also associated with regulating inhibitory dynamics in neural circuits (Wahis and Holt, 2021). Indeed, astrocytic α1ARs are implicated (alongside those expressed in interneurons) in increasing GABAergic neurotransmission.

Together, these studies suggest that Ca^2+^ elevations are a hallmark of astrocytic Gq-GPCR activation in the adult mouse hippocampus, whereby these dynamics directly impact synaptic activity. Indeed, astrocytic mGluR1/5 activation stimulates generation of mEPSCs and SICs in nearby neurons, thereby facilitating synaptic potentiation. Activation of astrocytic P2YR1s also seem to contribute to potentiating excitatory synapses, while astrocytic α1AR stimulation appears to regulate inhibitory neurotransmission. Overall, very little is known about the behavioral consequences of endogenous Gq-GPCR activity under physiological conditions (Table 1), prompting the requirement of *in vivo* techniques to fill this gap in the literature.

#### 4.1.2. Endogenous Gs-GPCRs

A number of different Gs-GPCRs are expressed in adult mouse hippocampal astrocytes, including the adenosine 2A receptor (A2AR) and βARs (Table 2). A2AR to be present at low levels in hippocampal astrocytes of adult mice (Batiuk et al., 2020). Furthermore, A2AR expression is known to increase upon neuroinflammatory brain insults whereby astrocytic A2AR activation increases cell proliferation and activation, indicating roles for A2AR in mediating astrogliosis, a known hallmark of AD (Boison et al., 2010; Lopes et al., 2021). Astrocytic A2ARs have indeed been linked to AD such that mRNA expression of A2AR in human hippocampal astrocytes is upregulated with disease progression (while expression is downregulated during normal aging) and strongly correlated with pathological amyloid beta and microtubule-associated protein tau accumulation (Orr et al., 2015). Furthermore, Orr et al. (2015) show that increased activity of astrocytic A2ARs across the whole brain impairs memory retention in a mouse model of AD. Functionally, activation of A2AR in cultured rat astrocytes is shown to increase cAMP levels (Murphy et al., 1991; Cristóvão-Ferreira et al., 2013). This typical Gs-coupled effect of cAMP elevations has also been associated with inhibiting nitric oxide production in astrocytes *via* A2AR activation (Brodie et al., 1998; Boison et al., 2010). Nitric oxide is an important retrograde messenger for LTP and LTD induction whereby extracellular nitric oxide plays a role in NMDAR-dependent LTP by action on postsynaptic Ca^2+^ channels (Paul and Ekambaram, 2011; Pigott and Garthwaite, 2016). We speculate that memory impairment upon activation of astrocytic A2ARs may be due to nitric oxide inhibition and subsequent impairment in LTP induction. However, the role of astrocytic A2AR activation under physiological conditions remains to be determined.

The presence of another group of Gs-GPCRs, βARs, has also been illustrated pharmacologically in hippocampal astrocytes of adult rodents (Salm and McCarthy, 1992; Gao et al., 2016; Oe et al., 2020). βAR activation is also known to mediate cAMP accumulation (Catus et al., 2011; Vardjan et al., 2014) leading to increased process branching in primary rat cultures (Vardjan et al., 2014), indicating a role for βARs in morphological plasticity. Astrocytic βAR activity in the mouse hippocampus is also shown to enhance LTP (Walker et al., 2017). In the rat hippocampus, astrocytic βAR antagonism prior to learning had no effect on short-term memory but significantly impaired recent and remote long-term memory, indicating activation of astrocytic βARs promotes memory consolidation (Gao et al., 2016). Furthermore, the role of βAR in long term memory appears to be mediated specifically by a β_2_AR-dependent increase in lactate release during learning. However, most studies do not discriminate between different βARs, limiting our understanding of subtype-specific effects.

Together, these studies suggest that endogenous A2AR and βAR activation in hippocampal astrocytes of the adult mouse mediate increased cAMP production with no effect on Ca^2+^ levels. However, Gs-GPCR effects on learning and memory appear to be receptor specific, whereby A2AR stimulation results in memory impairment in an AD mouse model (with so far unknown effects under physiological conditions), but βAR stimulation enhances memory.

#### 4.1.3. Endogenous Gi-GPCRs

Various Gi-GPCRs are also endogenously expressed in hippocampal astrocytes of adult mice (Table 3), including mGluR3, a group II mGluR (mGluR2/3). Strong expression profiles have been shown for mGluR3 in hippocampal astrocytes of adult mice by immunostaining (Sun et al., 2013) and scRNA-seq data (Batiuk et al., 2020). mGluR3 expression has also been observed in hippocampal astrocytes of the rhesus monkey, and was shown by immunoelectron microscopy to be located primarily at PAPs (Jin et al., 2018). This observation is congruent with findings by Lavialle et al. (2011) in adult rats, who showed that *in vivo* mGluR3 expression is largely confined to PAPs. Together, findings from Lavialle et al. (2011) and Jin et al. (2018) indicate that mGluR3 expression on hippocampal PAPs is conserved across species, although this is yet to be proven in adult mice. Another key Gi-coupled GPCR, the GABAb receptor (GABAbR), was also shown to be present in mouse hippocampal astrocytes by RNA sequencing and by pharmacological induction of Ca^2+^ signaling (Chai et al., 2017; Durkee et al., 2019; Batiuk et al., 2020). GABAbR expression has also been observed in PAPs of hippocampal astrocytes in the adult rat by immunostaining (Charles et al., 2003), but is yet to be observed in the adult mouse hippocampus.

Generally, activation of astrocytic Gi-GPCRs stimulates signaling pathways that elevate Ca^2+^ levels and inhibit cAMP production (Figure 1 and Table 3). Indeed, activation of astrocytic mGluR2/3, by a non-specific agonist in hippocampal mossy fiber circuits (DG-CA3) *in vivo*, elevates Ca^2+^ (Haustein et al., 2014). However, using the same agonist Chai et al. (2017) were unable to detect Ca^2+^ elevations in CA1 astrocytes. This could reflect regional differences for astrocytes in the hippocampus, concerning (i) differences in mGluR2/3 expression levels, (ii) interactions of mGluR2/with different G proteins or (iii) functional heterogeneity of the astrocytes themselves. For example, it was recently shown in HEK cells that Gi-GPCR-induced Ca^2+^ elevations are mediated by the Gi-βγ subunit, and that mobilization of the Gi-βγ subunit requires Gq co-activation (Pfeil et al., 2020). Thus, Gi-GPCR activation amplifies Gq-mediated Ca^2+^ responses *via* the PLC-IP_3_ signaling pathway – although the degree to which Gq co-activation is required for Gi-GPCR-induced Ca^2+^ signaling specifically in astrocytes, remains to be determined (Figure 1). Astrocytic GABAbR activation in mossy fiber circuits is also evokes Ca^2+^ events in parallel with mGluR2/3 (Haustein et al., 2014), with substantial impacts on neural circuit activity. For example, Covelo and Araque (2018) found that astrocyte GABAbR-dependent Ca^2+^ elevations are triggered *in situ* by interneuron stimulation in the mouse stratum radiatum and mediate IP_3_-dependent synaptic potentiation and depression. Furthermore, astrocyte GABAbR activity in mouse hippocampal slices mediated interneuron-induced potentiation of excitatory neurotransmission (Perea et al., 2016).

The effects of Gi-GPCR stimulation on cAMP levels is less studied than effects on Ca^2+^ levels. However, GABAbR activation is known to be negatively coupled to cAMP in rat primary astrocyte cultures (Patte et al., 1999). It has also been shown for hippocampal astrocytes that, quite counterintuitively, mGluR3 activity can potentiate cAMP elevations elicited by Gs-coupled βARs *via* Gi-βγ subunits as illustrated in Figure 1 (Tang and Gilman, 1991; Walker et al., 2017). Interestingly, mGluR3 potentiation of βAR-induced cAMP elevations disrupts βAR-mediated LTP induction and contextual fear learning, to a similar degree as direct βAR antagonism. It is suggested that accumulation of astrocytic cAMP has this antagonistic effect through subsequent activation of presynaptic adenosine receptors, ultimately attenuating LTP induction. Indeed, βARs and mGluR3 are co-localized on hippocampal astrocytes and are therefore optimally located to cooperate in regulating cAMP signaling, synaptic potentiation, and memory (Walker et al., 2017).

Cannabinoid receptor 1 (CB1R) is a GPCR thought to preferentially couple to Gi proteins. However, depending upon agonist availability, CB1R ligand binding stabilizes distinct receptor conformations thereby favoring coupling to either Gi or Gq proteins (Lauckner et al., 2005; Tables 2, 3). Particular agonists (for example WIN55, 212-2) stabilize CB1R confirmations in favor of Gq-coupling (Lauckner et al., 2005). However, disentangling the contributions of Gi- and Gq-coupled CB1R pathways requires further detailed investigation. CB1R expression has been observed in astrocytes of the adult mouse hippocampus by immunostaining, electron microscopy, and by functional responses to application of endogenous and synthetic agonists (Navarrete and Araque, 2008; Han et al., 2012; Gutiérrez-Rodríguez et al., 2018). CB1R activation is known to stimulate the PLC-IP_3_ signaling pathway and mediate Ca^2+^ release from intracellular stores (Navarrete and Araque, 2008), which could be due to Gi- and/or Gq-coupling, as both are shown to mediate IP_3_-dependent Ca^2+^ signaling by action of the Gi-βγ and Gq-α subunits respectively (Zeng et al., 2003; Figure 1). These CB1R-evoked Ca^2+^ elevations have been shown to increase extracellular D-serine levels (Robin et al., 2018) and induce NMDAR-dependent SICs in nearby excitatory neurons in the mouse CA1 (Navarrete and Araque, 2008), indicating an important role for astrocytic CB1Rs in synaptic potentiation in the mouse hippocampus. Indeed, temporal coincidence of CB1R-evoked Ca^2+^ signaling in astrocytes with postsynaptic activity is shown to induce LTD at synapses close to the source of endocannabinoid, but LTP at relatively more distant synapses in mouse hippocampal slices (Navarrete and Araque, 2010; Min and Nevian, 2012; Gómez-Gonzalo et al., 2015). It has also been shown exogenous cannabinoid stimulation of astrocytic CB1Rs *in vivo* evokes LTD and spatial working memory impairments in mice by activation of NMDAR and internalization of AMPAR in CA1 post-synaptic neurons (Han et al., 2012). Together with the observation that total CB1R deletion in hippocampus impairs memory extinction (Marsicano et al., 2002), the above studies show that CB1Rs in hippocampal astrocytes have a physiological role in regulating memory retention/extinction processes.

In summary, endogenously expressed astrocytic CB1R, GABAbR and mGluR2/3 evoke Ca^2+^ elevations by Gi-βγ subunit stimulation of the PLC-IP_3_ signaling pathway, but it remains unclear whether Gi-βγ-mediated Ca^2+^ elevations can occur independently of Gq-GPCR activity. Endogenous Gi-GPCRs also downregulate cAMP production in astrocytes *via* Gi-α subunit activity. However, co-activation of mGluR3 with βAR seems to potentiate βAR-Gs-mediated elevation of cAMP levels. A number of studies also show astrocytic Gi-GPCR activity impacts synaptic potentiation and memory processes.

## 5. Comparative assessment of endogenous GPCRs and DREADDs

### 5.1. Endogenous Gq-GPCRs vs. Gq-DREADDs

A number of studies have directly compared endogenous Gq-GPCR activation to that of Gq-DREADDs in astrocytes. Here, we integrate these studies with those investigating either endogenous Gq-GPCRs or Gq-DREADDs individually, to obtain more insight into the astrocytic response characteristics, and the synaptic and behavioral effects of endogenous Gq-GPCR versus Gq-DREADD stimulation.

It has been shown that the amplitude and temporal characteristics of evoked Ca^2+^ responses in hippocampal astrocytes are comparable for Gq-DREADD and endogenous Gq-GPCR activation (Agulhon et al., 2013; Durkee et al., 2019; Vaidyanathan et al., 2021). Indeed, endogenous mGluR1/5 stimulation evokes Ca^2+^ elevations in the mouse hippocampus with stimulus-strength response heterogeneity (Xie et al., 2012), and similarly, astrocytic Gq-DREADD stimulation in the cortex produces multi-peak Ca^2+^ signals at low CNO concentrations (0.2 mg/kg) and plateaued responses at higher doses (1 mg/kg) (Bonder and McCarthy, 2014). Furthermore, chronic stimulation of endogenous Gq-GPCRs is associated with receptor desensitization (Rajagopal and Shenoy, 2018; Dyer-Reaves et al., 2019). This aligns with observations that Gq-DREADD activation (due to chronic ligand availability) silences evoked Ca^2+^ responses after just a few minutes of repeated stimulation (Vaidyanathan et al., 2021). Stimulation of astrocytic Gq-DREADDs has yet to illustrate the transient, single peak Ca^2+^ elevations which can be observed upon endogenous α1AR stimulation (Srinivasan et al., 2015; Oe et al., 2020). It is unclear at present why these α1AR effects cannot be replicated by Gq-DREADDs (hM3Dq), however it could be a reflection of the systemic ligand delivery system resulting in chronic ligand availability, as opposed to acute spikes in endogenous, locally released, ligand concentrations (Claes et al., 2022), or due to underlying functional differences between endogenous α1ARs and the muscarinic-derived DREADDs.

**FIGURE 2:**
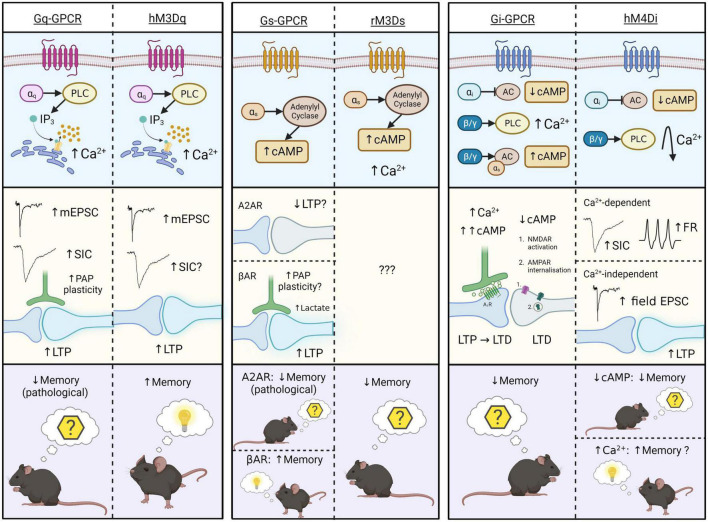
Schematic representation of intracellular **(top)**, synaptic **(middle)**, and behavioral **(bottom)** effects of endogenous Gq-, Gs-, and Gi-GPCR stimulation in astrocytes, and that of corresponding Gq-, Gs-, and Gi-DREADDs (hM3Dq, rM3Ds, and hM4Di). Astrocytic Gq-GPCRs/hM3Dq activation **(left)** evokes Ca^2+^ elevations *via* the PLC-IP_3_ signaling pathway. This increases mEPSCs, SICs (conflicting evidence for hM3Dq), LTP and PAP plasticity at synapses. Only one study illustrates memory-related behavioral effects of Gq-GPCR activation in astrocytes – in pathological condition (AD) Gq-GPCR activation mediated memory impairment, while hM3Dq activations mediate memory enhancement under physiological conditions. Astrocytic Gs-GPCR and rM3Ds activation **(centre)** evokes cAMP elevations through stimulation of adenylyl cyclase (AC), and rM3Ds activations evoke Ca^2+^ elevations through unknown mechanisms. We speculate that astrocytic A2AR activation impairs LTP. Astrocytic βAR activation is shown to mediate morphological plasticity, possibly including the PAPs, and lactate release, thereby stimulating LTP at synapses. These synaptic effects have not been shown for the Gs-DREADD hM3Ds, illustrating a major gap in the literature. Behavioral effects of astrocytic A2AR and rM3Ds activation is on memory extinction, however βAR stimulation evokes memory enhancement. Astrocytic Gi-GPCR and hM4D activation **(right)** evokes reductions in cAMP levels by inhibition of AC. However, the endogenous Gi-GPCR mGluR3 can also elevate cAMP levels by action of the βγ subunit, through potentiation of Gs-coupled elevations. Stimulation of Gi-GPCRs evokes Ca^2+^ elevations *via* the PLC signaling pathway. Corresponding synaptic effects of cAMP- and Ca^2+^-elevations include synaptic potentiation and depression, whereby cAMP accumulation leads to activation of presynaptic adenosine 1 receptors (A_1_R) and LTP disruption. Downregulated cAMP by astrocytic Gi-GPCRs mediate post-synaptic NMDAR activation and AMPAR internalization, thereby evoking LTD. Together these intracellular and synaptic effects, evoked by lowering astrocytic cAMP-levels, have a negative effect on memory retention. Astrocytic Gi-DREADD (hM4Di) activation is also shown to evoke cAMP reductions and thereby inhibition of fear learning. In addition, other studies reported that astrocytic hM4Di activation can also evoke transient Ca^2+^ elevations that return to levels below baseline thereafter. These Ca^2+^ effects are shown to increase frequency of SICs, firing rates (FR) and LTP in nearby neurons, potentially having a positive effect on memory. The final effect of Gi-DREADD signaling on memory therefore seems to depend on the net outcome of cAMP and Ca^2+^ effects. Figure created with www.biorender.com.

Direct comparisons by Durkee et al. (2019) show that stimulation of Gq-DREADDs or endogenous Gq-GPCRs both evoke Ca^2+^ elevations by PLC-IP_3_ signaling cascades in the somata and processes of hippocampal astrocytes (Figure 2). This observation is verified by other investigations of Gq-DREADDs (Chai et al., 2017; Vaidyanathan et al., 2021) and endogenous Gq-GPCRs (Xie et al., 2012; Srinivasan et al., 2015; Oe et al., 2020; Lia et al., 2021; Lawal et al., 2022). Interestingly, the specific IP_3_R subtype mediating astrocytic Ca^2+^ fluctuations may indicate the subcellular origin of these fluctuations (Sherwood et al., 2021), thereby influencing the impact of astrocytic Ca^2+^ elevations on synaptic function and behavior. The predominant narrative is that IP_3_R2 is responsible for Gq-GPCR Ca^2+^ elevations, with early reports showing that genetic knock-out of IP_3_R2 abolished Ca^2+^ transients in astrocytes, without impacting *SIC* generation, neuronal ionotropic glutamate receptor activation (Fiacco et al., 2007), neuronal Ca^2+^ activity (Fiacco et al., 2007; Petravicz et al., 2008), spontaneous or evoked EPSCs (Petravicz et al., 2008; Agulhon et al., 2010) or long-term synaptic plasticity mechanisms (Petravicz et al., 2014). However, these studies do not account for Ca^2+^ signaling in PAPs, which likely has a more significant impact on neuronal function than somatic Ca^2+^ fluctuations (Haustein et al., 2014; Srinivasan et al., 2015; Sherwood et al., 2021). Indeed, more recent studies indicate that PAP Ca^2+^ activity relies primarily upon IP_3_R2-independent mechanisms (Srinivasan et al., 2015; Liu J.-H. et al., 2022). For example, IP_3_R1 and IP_3_R3 are shown to contribute to Ca^2+^ signaling in PAPs, alongside IP_3_R2 (Sherwood et al., 2017). As a result, it could be that IP_3_R2 knockout does not significantly impact neuronal function due to the compensating ability of IP_3_R1 and IP_3_R3 in PAPs (Bazargani and Attwell, 2016). Accordingly, Sherwood et al. (2021) speculate that astrocytic IP_3_ receptors have subtype-specific cellular distributions according to their functional properties, however this has yet to be empirically assessed. The predominance and relative importance of each IP_3_R subtype within PAPs remains an interesting open question – one which could contribute to our understanding of how endogenous Gq-GPCR and Gq-DREADD subcellular localization and evoked Ca^2+^ signaling impacts downstream synaptic and behavioral effects.

A major, unresolved issue on Gq-DREADD activation effects in astrocytes, is their Ca^2+^-dependent effects on neurons. Endogenous Gq-GPCR-mediated Ca^2+^ activity is consistently shown to impact synaptic activity in the adult mouse hippocampus (Figure 2). However, early MrgA1 (Fiacco et al., 2007; Agulhon et al., 2010) and IP_3_R2 knockout studies (Fiacco et al., 2007; Petravicz et al., 2008, 2014; Agulhon et al., 2010; Bonder and McCarthy, 2014) contradict the hypothesis that astrocytic Gq-GPCR activation and subsequent Ca^2+^ elevations impact synaptic activity and plasticity mechanisms. To our knowledge, the source of this discrepancy is unknown, but likely represents (i) the uncharacterized complexity in the spatiotemporal dynamics of evoked Ca^2+^ signaling (e.g., subcellular localization of GPCRs in PAP domains), (ii) interactions between receptors [e.g., synergistic effects of mGluR3 (Gi) with βAR (Gs)] and (iii) the presence (or absence) of functionally relevant neuromodulators (e.g., TNFα). It is also worth noting that the inability of some studies to detect synaptic changes upon astrocytic Gq-DREADD stimulation is not strong evidence of absence for this hypothesis – rather, it simply shows that synaptic effects were not observed in this case. Indeed, there is substantial evidence in favor of the hypothesis that Ca^2+^ elevations evoked both by endogenously expressed astrocytic Gq-GPCRs, and Gq-DREADDs, alter synaptic activity in the adult mouse hippocampus (Figure 2).

At a behavioral level, Gq-DREADDs and endogenous Gq-GPCRs in hippocampal astrocytes are shown to impact memory (Adamsky et al., 2018; Reichenbach et al., 2018). However, while astrocytic Gq-DREADD activation enhances memory (Adamsky et al., 2018), endogenous astrocytic Gq-GPCR activation contributes to memory impairment in an AD mouse model (Reichenbach et al., 2018). While these results appear contradictory (Figure 2), the severity of pathology in this model impedes direct comparisons between endogenous and synthetic receptors. However, it could be that these maladaptive effects on memory are induced upon chronic, pathological Gq-GPCR activation in astrocytes, whereas the physiological activations have memory enhancing effects, similar to those observed by Adamsky et al. (2018). The impact of endogenous Gq-GPCR activation in astrocytes in the healthy brain is yet to be observed, and therefore the degree to which the behavioral consequences of endogenous and synthetic receptor stimulation are comparable in the healthy brain remains an open question.

Overall, these results suggest that improved methodologies and theoretical understanding has generated a loose consensus that astrocytic Gq-coupled receptor stimulation in the adult mouse hippocampus elevates Ca^2+^ levels and potentiates synaptic activity (Figure 2). However, it remains unclear whether subsequent behavioral effects are equally comparable (Figure 2).

### 5.2. Endogenous Gs-GPCRs vs. Gs-DREADDs

To our knowledge, Orr et al. (2015) is the only study to directly compare Gs-GPCR and Gs-DREADD activation effects in astrocytes. Activation of endogenous, and synthetic Gs-GPCRs in adult mouse hippocampal astrocytes, mediates increased cAMP levels without impacting intracellular Ca^2+^ concentrations (Orr et al., 2015). This comparison is substantiated by other studies, on hippocampal astrocytes, showing cAMP elevation upon Gs-DREADD activation (Oe et al., 2020) and upon Gs-coupled βAR activation *in vitro* (Catus et al., 2011) and *in situ* (Walker et al., 2017). Activation of endogenous βARs stimulates astrocyte protrusions *in vitro* (Vardjan et al., 2014), lactate release (Zhou et al., 2019) and LTP induction (Walker et al., 2017) at synapses. Furthermore, astrocytic A2AR activity is thought to impair LTP induction *via* inhibition of nitric oxide production (Boison et al., 2010). However, while the intracellular effects of stimulating endogenous Gs-GPCRs and Gs-DREADDs in astrocytes are comparable, synaptic effects of endogenous Gs-GPCR activity have not yet been replicated by Gs-DREADD experiments (Figure 2), representing a major gap in the literature yet to be addressed.

Astrocytic Gs-DREADD activation has also been shown to elicit Ca^2+^ elevations (Chai et al., 2017; Nagai et al., 2021), however an effect of endogenous Gs-GPCRs on calcium elevation has not been reported so far. Accordingly, we speculate that Gs-DREADD-evoked Ca^2+^ elevations are DREADD-specific, and due to promiscuous binding to Gq proteins, given the relative structural homogeneity between rM3Ds and hM3Dq.

At the behavioral level, astrocytic Gs-DREADD stimulation is associated with impaired memory consolidation (Orr et al., 2015), in line with our understanding of astrocytic A2AR activation effects on memory (Boison et al., 2010; Paul and Ekambaram, 2011; Orr et al., 2015). However, activation of βAR is shown to promote long term memory consolidation in rats (Gao et al., 2016). Hence, while both βAR and A2AR evoke cAMP elevations (Catus et al., 2011; Orr et al., 2015), the effects of βAR activation on memory oppose that of A2AR. This discrepancy could be explained by manipulating A2AR expression in whole brain (Orr et al., 2015) compared to just in hippocampus (Gao et al., 2016), or the presence of divergent downstream mechanisms eliciting distinct synaptic effects (Figure 2). Given this, further receptor-specific research is required to uncover the precise nature of signaling pathways downstream of cAMP elevations, including the potential influence of different receptor expression levels, subcellular localization and interactions with Gi and Gq proteins.

### 5.3. Endogenous Gi-GPCRs vs. Gi-DREADDs

Finally, we compare the intracellular signaling mechanisms, synaptic and behavioral consequences of endogenous Gi-GPCR and Gi-DREADD activation in astrocytes. We will first discuss these effects in relation to cAMP, followed by those mediated by Ca^2+^ signaling.

Endogenously expressed Gi-GPCRs in astrocytes are characterized by their inhibitory effects on adenylyl cyclase activity and subsequent reductions to cAMP levels (Patte et al., 1999; Han et al., 2012; Walker et al., 2017), an effect that has been replicated by Gi-DREADDs in the rodent hippocampus (Jones et al., 2018). This Gi-DREADD-mediated attenuation of intracellular cAMP signaling is also shown to impair stress-enhanced fear learning (Jones et al., 2018). Impaired remote memory retrieval has also been observed upon astrocytic Gi-DREADD activation (Kol et al., 2020), although the contribution of cAMP was not measured in this study. Similar behavioral effects were observed upon stimulation of endogenous mGluR3 in the mouse hippocampus by Walker et al. (2017), which, in contrast to the effects of Gi-DREADD activation, were associated with cAMP upregulation due to interactions between the mGluR3 βγ subunit and coactivated, Gs-coupled βARs (Walker et al., 2017). Testing whether this effect is conserved in DREADDs signaling would further strengthen the notion that DREADDs can accurately mimic their endogenous counterparts. However, receptor expression levels and sub-cellular localization may impact the ability of Gi-DREADDs to interact with other receptors, limited their ability to replicate this particular effect. Overall, there seems to be a shared effect of endogenous and synthetic astrocytic Gi-coupled receptors in that both impair contextual and stress-enhanced fear memory and remote retrieval, however the precise role of cAMP in this remains unclear (Figure 2).

Gi-DREADD activation in astrocytes has also been shown to elicit Ca^2+^-independent potentiation of excitatory CA1 synapses (Van Den Herrewegen et al., 2021; Figure 2). These potentiating effects on excitatory synapes, while Ca^2+^-independent and thus possibly cAMP-related, are expected to cause memory enhancement – however Ca^2+^-independent memory enhancement has not been empirically proven for endogenous Gi-GPCRs or Gi-DREADDs in astrocytes. As a result, it is unclear if Ca^2+^-independent, Gi-DREADD-evoked synaptic potentiation is (i) due to cAMP downregulation or other mechanisms, (ii) can evoke memory enhancement, and (iii) whether these effects mimic endogenous processes or are Gi-DREADD-specific. The disparity between synaptic and behavioral effects of Gi-DREADD and endogenous Gi-GPCR activation in astrocytes indicate that future studies are needed to further establish the downstream effects of cAMP modulation by astrocytic Gi-GPCRs and the precise manner in which these effects diverge from those elicited by Gi-DREADDs. Increased understanding of brain region- and receptor-specific differences, expression levels and interactions with other G proteins will likely aid in determining how Gi-coupled receptor activation modulates cAMP and memory processes.

In accordance with the literature outlining the effects of endogenous Gi-GPCR stimulation, some studies show that Gi-DREADDs evoke Ca^2+^ elevations (Haustein et al., 2014; Durkee et al., 2019; Kol et al., 2020; Vaidyanathan et al., 2021), while others indicate that they do not (Chai et al., 2017; Nam et al., 2019; Van Den Herrewegen et al., 2021). However, as shown in Figure 2, both Gi-GPCRs and Gi-DREADDs have been shown to elicit Ca^2+^ elevations *via* the PLC-IP_3_ signaling pathway (Navarrete and Araque, 2008; Haustein et al., 2014; Covelo and Araque, 2018; Kol et al., 2020; Vaidyanathan et al., 2021). However, while astrocytic Gi-DREADD activation in mouse hippocampus evokes moderate reductions in astrocytic Ca^2+^ levels after initial transient peaks (Kol et al., 2020), this has not been observed upon endogenous Gi-GPCR stimulation in astrocytes (Figure 2). The finding that at least some Gi-GPCRs require co-activation of Gq-GPCRs to evoke Ca^2+^ elevations by action of the βγ subunit on the PLC-IP_3_ signaling pathway (Pfeil et al., 2020) could explain why some studies show robust Gi-evoked Ca^2+^ signaling, while others show little to no effect – if constitutive Gq activity is low, this could theoretically impact the capacity of Gi-GPCRs to stimulate Ca^2+^ signaling. Whether or not Gq-dependence is also a feature of Gi-GPCR and Gi-DREADD-mediated Ca^2+^ activity specifically in mouse hippocampal astrocytes has yet to be determined. However, further investigation of this mechanism in endogenous Gi-GPCRs and Gi-DREADDs could clarify the degree to which Ca^2+^ signaling mechanisms in Gi-DREADDs mimic the endogenous process even further.

Taken together, these studies show that astrocytic Gi-DREADDs mimic astrocytic Gi-GPCR activity in many aspects. This includes cAMP downregulation, PLC-IP_3_-induced Ca^2+^ elevations (in some but not all instances), synaptic potentiation and memory impairment (Figure 2). However, there remain some distinguishing factors. It has not yet been shown whether endogenous Gi-GPCRs in astrocytes evoke Ca^2+^ elevations which share the same temporal profile as observed upon Gi-DREADD stimulation. Unlike endogenous Gi-GPCRs in astrocytes, astrocytic Gi-DREADDs can evoke increases to cAMP levels, as is shown to occur upon co-activation of mGluR3 and Gs-coupled βAR. Furthermore, while activation of astrocytic Gi-DREADDs elicits synaptic effects indicative of memory enhancement, this has not yet been shown for endogenous Gi-GPCRs (Figure 2).

## 6. Concluding remarks

The most frequently studied GPCR subtype, Gq, seems to show a high level of congruence between DREADD-induced and endogenous GPCR effects. This is particularly true in relation to downstream mechanisms elicited by their activation. Indeed, PLC-IP_3_ signaling mechanisms evoke Ca^2+^ elevations with similar physical and temporal properties upon activation of both endogenous Gq-GPCRs and Gq-DREADDs. Subsequent effects on neuronal activity are also comparable, indicating DREADDs accurately mimic astrocytic GPCR-mediated intracellular and synaptic effects in the adult mouse hippocampus. Limited available data show memory-related behaviors were not comparable; however, this likely relates to difficulties directly comparing behaviors between physiological and pathological conditions. With respect to Gs-coupled receptors, activation of Gs-DREADDs appears to replicate intracellular cAMP elevations associated with endogenous A2AR and βAR stimulation in astrocytes. However, the behavioral consequences of Gs-DREADD stimulation in astrocytes mimic only those observed upon βAR activation, opposing that of A2AR. This is likely the result of diverging downstream signaling pathways and synaptic effects which require further region-specific characterization. Similarly, while activation of Gi-DREADDs mimics that of endogenous Gi-GPCRs in some ways, there are a number of exceptions. Both appear to inhibit cAMP production, inconsistently evoke Ca^2+^ elevations, and induce memory impairment. However, the ability of mGluR3 to potentiate cAMP has not yet been reported in Gi-DREADD studies, while the Gi-DREADD-mediated enhancement of synaptic potentiation and memory acquisition has not yet been shown for endogenously expressed GPCRs.

Mounting evidence suggests that the relationship between intracellular Ca^2+^ elevations, synaptic changes and behavior is not a black and white binary, but rather depends upon activation of specific receptor subtypes, likely at specific sub-cellular locations and timepoints – the complexity of which is yet to be fully understood (Shigetomi et al., 2008; Bazargani and Attwell, 2016). With this in mind, disparities between the effects of astrocytic DREADDs and their endogenous counterparts could be due to a variety of factors. First, we must consider that although DREADDs are often assumed to bind specifically to Gq-, Gs- or Gi-proteins, it is likely that they mimic downstream mechanisms specific to their respective parent muscarinic receptors, which are not necessarily Gq-, Gs-, or Gi-specific. While this question of DREADD specificity may complicate the interpretation of experiments aiming to elucidate the mechanisms of specific G-protein activation, it does increase the degree to which DREADDs accurately mimic endogenous GPCR activity. Indeed, these considerations may explain observed functional differences between DREADDs and some endogenous receptors. That is, although DREADDs aim to simulate generalized downstream mechanisms of Gq-, Gs-, and Gi-GPCR activation, imperfect reflections of other receptor types such as the α1AR, may simply reflect their muscarinic lineage. Thus, a certain degree of functional distinction between DREADDs and endogenous GPCRs is likely inherent to experimental technique itself, without nullifying its utility. Furthermore, it is known that current methods cannot accurately mimic endogenous spatiotemporal expression (McNeill et al., 2021; Shen et al., 2021) or excitation patterns (Nimmerjahn and Bergles, 2015; Sherwood et al., 2021). Indeed, the stochastic spatial distribution of DREADD expression likely does not reflect endogenous distributions of GPCRs. By extension, this could impact synaptic and behavioral effects of astrocytic DREADD stimulation, limiting their equivalence to the effects of endogenous GPCR activity. Incorporation of morphological examinations in future DREADD experiments in astrocytes could shed light on this issue. Furthermore, the chronic availability of DREADD ligands could be contributing factor to inconsistent results between DREADD-mediated effects and those evoked by acute and local stimulation of endogenous GPCRs, given differing temporal dynamics of receptor stimulation (Claes et al., 2022).

This comparative assessment suggests that controversies arising from astrocytic DREADD experiments are largely due to the sheer complexity of the system at hand. Increased research in recent years has, to a certain extent, clarified the physiological plausibility of DREADDs in the study of astrocytic GPCRs and their roles at the synapse and in memory. All GPCR subtypes assessed here show some degree of similarity between effects of DREADDs and endogenous GPCRs. Furthermore, it seems that a higher degree of consistency between DREADDS and endogenous receptors is achieved with increased research. We therefore expect the current understanding of GPCR activity in astrocytes to improve accordingly. Manipulating astrocyte activity using DREADDs can and has illustrated important roles for these cells at synapses, within larger networks and in behavior. This method, in combination with the study of endogenous GPCRs, can therefore provide indications as to the molecular and cellular mechanisms mediating synaptic and behavioral effects. Overall, the use of DREADDs in astrocytes is at least, hypothesis generating. But, at best, can provide us unprecedented access to the intersection between astrocytic GPCR physiology and synaptic function and behavior.

## Author contributions

SL contributed to the conceptualization, manuscript selection and reading, visualization, and writing of the draft. AM contributed to the manuscript selection and editing. MV contributed to the conceptualization, review, editing, and supervision. All authors contributed to the article and approved the submitted version.
